# Clinical Characteristics of COVID-19 Patients with and without Diabetes in Wuhan Red Cross Hospital

**DOI:** 10.1155/2020/1652403

**Published:** 2020-08-10

**Authors:** Guozhen Li, Qin Deng, Jiali Feng, Fang Li, Nian Xiong, Qiong He

**Affiliations:** ^1^Department of Gastroenterology, Wuhan Red Cross Hospital, Wuhan, China; ^2^Department of Neurology, Union Hospital, Tongji Medical College, Huazhong University of Science and Technology, Wuhan, China

## Abstract

**Background:**

Since the end of December 2019, novel coronavirus- (SARS-CoV-2-) infected pneumonia (COVID-19) has spread rapidly in China. The study was designed to describe the clinical characteristics of COVID-19 patients with and without diabetes in Wuhan.

**Methods:**

199 COVID-19 patients were admitted to Wuhan Red Cross Hospital in China from January 24^th^ to March 15^th^. The cases were divided into the diabetic and nondiabetic groups according to the history of taking antidiabetic drugs or by plasma fasting blood glucose level at admission, and the differences between the groups were compared. A multivariable analysis of independent risk factors for in-hospital death was performed.

**Results:**

Among 199 COVID-19 patients, 76 were diabetic and 123 were nondiabetic. Compared with nondiabetics, patients with diabetes were older and had high levels of fasting plasma glucose (FPG), D-dimer, white blood cells, blood urea nitrogen (BUN), and total bilirubin (TBIL) and lower levels of lymphocytes, albumin and oxygen saturation (SaO_2_), and higher mortality (*P* < 0.05). The two groups showed no difference in clinical symptoms. Diabetes (odds ratio (OR): 10.816, 95% CI (confidence interval): 1.895-61.741, *P* = 0.007), higher level of D-dimer at admission (OR: 1.094, 95% CI: 1.017-1.178, *P* = 0.016), and a lymphocyte count < 0.6 × 10^9^/L at admission (OR: 7.609, 95% CI: 1.087-32.049, *P* = 0.006) were associated with increasing odds of death. Antidiabetic drugs were associated with decreasing odds of death. Treatment with low molecular weight heparin was not related to odds of death.

**Conclusion:**

The mortality rate of COVID-19 patients with diabetes was significantly higher than those without diabetes. Diabetes, higher level of D-dimer, and a lymphocyte count < 0.6 × 10^9^/L at admission were the risk factors associated with in-hospital death.

## 1. Introduction

Since the end of December 2019, novel coronavirus- (SARS-CoV-2-) infected pneumonia (COVID-19) has rapidly spread throughout China and around the world [1–4]. The virus was named SARS-CoV-2 by the International Committee on Taxonomy of Viruses, and the disease caused by the virus was termed COVID-19 [5]. The high infectivity of COVID-19 resulted in a rapid increase of new cases. So far, large-scale analyses have reported the clinical presentation and clinical outcomes of patients with COVID-19 [6, 7]. Many studies have focused on the clinical characteristics and outcomes of COVID-19 patients with diabetes.

Diabetic patients have been identified at a high risk of respiratory infections [8]. Studies have reported the relationship between blood glucose levels and the clinical course of severe acute respiratory syndrome (SARS) [9]. Some meta-analyses confirmed that diabetes increased the mortality of COVID-19 patients [10–12]. In this study, the aim was to determine clinical symptoms, laboratory findings, and mortality of patients with diabetes and patients without diabetes in COVID-19 and to report on any difference.

## 2. Methods

### 2.1. Patients

The retrospective study was approved by the ethics committee of Wuhan Red Cross Hospital (no. 2020022). All COVID-19 patients admitted to Wuhan Red Cross Hospital from January 24^th^ to March 15^th^ were enrolled. During this period, Wuhan Red Cross Hospital became a special designated hospital for the treatment of COVID-19 patients.

### 2.2. Definitions

COVID-19 was confirmed by detecting SARS-CoV-2 RNA in throat swab samples using a virus nucleic acid detection kit according to the manufacturer's protocol (Shanghai BioGerm Medical Biotechnology Co., Ltd.). All of COVID-19 patients were admitted to the general fever ward excluding the intensive care unit. The cases were divided into the diabetic and nondiabetic groups according to the history of taking antidiabetic drugs or by plasma fasting blood glucose level at admission.

### 2.3. Data Collection

The case report form of COVID-19 was designed to document primary clinical data regarding previous medical history, clinical symptoms, laboratory findings, and clinical outcomes from medical records. The following information was extracted for each patient: medical history, gender, age, clinical outcomes, and signs, symptoms, oxygen saturation, and laboratory findings at admission.

### 2.4. Data Analysis

Continuous data were described as median with interquartile range (IQR), and categorical data as percentages. *χ*^2^ test for categorical data and Mann–Whitney *U* test for continuous data were used to compare variables between the groups. A multivariable analysis of independent risk factors for in-hospital death was performed. SPSS Statistics version 16.0 software was used for statistical analysis. *P* < 0.05 was examined statistically significant.

## 3. Results

### 3.1. Baseline Characteristics

199 patients with COVID-19 pneumonia were included in our study (Table 1). Among them, 76 were diabetic and 123 were nondiabetic. The median age of COVID-19 patients with diabetes was 67 years (IQR: 61~78), which was significantly higher than that of patients without diabetes (IQR: 47~68, *P* ≤ 0.001). The onset of initial symptom to hospital admission in the patients with diabetes group was 10 days (IQR: 5 ~ 14), while the patients without diabetes group was 12 days (IQR: 7 ~ 20, *P* = 0.036). Duration of hospital stays was similar for both groups (*P* = 0.968). Of 199 patients, fever (74.4%), cough (66.8%), dyspnea (26.8%), and fatigue (25.1%) were the most common symptoms, while diarrhea (3%), vomiting (2.5%), and dizziness (1%) were scarce. However, there was no statistically significant difference in all of clinical symptoms between the diabetic and nondiabetic patients with COVID-19 pneumonia.

### 3.2. Laboratory Findings

The majority of patients with diabetes had abnormalities of D-dimer and fasting plasma glucose (FPG) at admission as described in Table 2. COVID-19 patients with diabetes had a higher level of fasting plasma glucose (*P* ≤ 0.001), D-dimer (*P* ≤ 0.001), white blood cells (*P* = 0.011), total bilirubin (*P* = 0.030), and blood urea nitrogen (*P* = 0.022) and lower level of oxygen saturation (*P* ≤ 0.001), lymphocyte (*P* = 0.036), and albumin (*P* = 0.002).

### 3.3. Regression Analysis

In the multivariable logistic regression model, we included 199 patients with complete data for all variables (181 survivors and 18 nonsurvivors). We found that diabetes (OR: 10.816, 95% CI: 1.895-61.741, *P* = 0.007), higher level of D-dimer at admission (OR: 1.094, 95% CI: 1.017-1.178, *P* = 0.016), and a lymphocyte count < 0.6 × 10^9^/L at admission (OR: 7.609, 95% CI: 1.087-32.049, *P* = 0.006) were associated with increasing death risk. Antidiabetic drugs were associated with decreasing odds of death. Treatment with low molecular weight heparin was not related to odds of death (Table 3).

#### 3.3.1. Clinical Outcome

**Figure 1:**
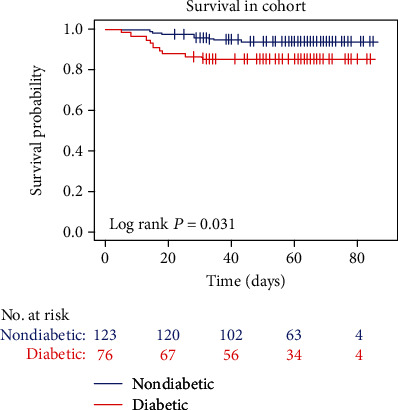
Survival curve in patients with COVID-19 pneumonia with and without diabetes.

There were 18 reported deaths in the patients with COVID-19 (Table 1). Significant difference in mortality was found between patients with diabetes and without diabetes (*P* = 0.036). 11 of 76 patients with COVID-19 with diabetes died (14.5%), while 7 of 123 patients with COVID-19 without diabetes died (5.7%). Diabetes seems to increase the risk of death in patients with COVID-19 pneumonia (log rank *P* = 0.031) (Figure 1).

## 4. Discussion

COVID-19 has received more attention compared to other causes of pneumonia, especially after the emergence of SARS and MERS. In certain risk factors, clinical manifestations, and clinical outcomes, COVID-19 was similar to SARS and MERS. The comorbidity of diabetes was reported as an independent predictor for morbidity and death in SARS-infected patients [9]. In our study, 11 (14.5%) patients with COVID-19 pneumonia with diabetes died, while 7 (5.7%) patients with COVID-19 pneumonia without diabetes died (*P* = 0.036). Diabetes was associated with increasing odds of death. Antidiabetic drugs were associated with decreasing odds of death. In this study, 199 COVID-19 patients were divided into the diabetic and nondiabetic groups. We compared the clinical features, laboratory findings, and clinical outcome between the two groups.

The study found similar proportions of male and female patients in COVID-19 with and without diabetes. The COVID-19 patients with diabetes had an older age than those without diabetes. According to previous reports, the old patients were at high risk of mortality in MERS and SARS [13, 14]. Recent studies had confirmed that death in patients with COVID-19 was associated with increased age [15]. Almost 90% COVID-19 death cases were than 70 years old in Italy [16]. In our study, patients with diabetes with COVID-19 were older than patients without diabetes. Thus, this increased their mortality also for their age.

In our cohort, patients with diabetes had a lower level of lymphocyte than those without diabetes. On admission, lymphocyte count < 0.6 × 10^9^/L was associated with increasing death risk. In previous studies, lymphocytopenia was common in the critical MERS patients, and this might be caused by lymphocyte apoptosis [17, 18]. Yang et al. reported that lymphocytopenia had an incidence of more than 80% in critically ill COVID-19 patients [19]. As for the specific damage by SARS-CoV viral particles, lymphocytopenia was common in critically ill COVID-19 patients [20]. In our study, the diabetic COVID-19 patients had only mild lymphocytopenia at admission, and the severity of lymphocytopenia could reflect the disease progression.

Higher level of D-dimer at admission was associated with increased odds of death in our study. D-dimer levels were quite different between the diabetic and nondiabetic groups (*P* ≤ 0.001). For patients with diabetes with COVID-19, D-dimer levels increased dramatically. D-dimer is an activation marker of fibrinolysis. Some studies have shown that D-dimer was a robust prognostic predictor in pneumonia and sepsis patients [21, 22]. D-dimer is a marker of mortality in patients admitted to the emergency department with suspected infection [22]. Recent studies found a higher level of D-dimer in the death group than in the survival group, and the COVID-19 patients with an increased D-dimer at admission were at high-risk death [7, 15, 23]. We found patients with COVID-19 with a higher level of D-dimer at admission, especially those with diabetes, are significantly associated with the risk of death. Treatment with low molecular weight heparin was not related to odds of death. Magro et al. reported that severe COVID-19 infection was associated with microvascular injury and thrombosis [24]. The need for further clinical trials using anticoagulants to determine whether the application of anticoagulants is effective.

Currently, few public studies have shown the specific cause of high mortality in patients with COVID-19 with diabetes. Diabetic patients have been identified at a high risk of respiratory infections. Many changes occurred in the immune system of diabetic patients. There were significant changes in humoral and cell-mediated immune function, especially related to abnormal pulmonary function.

In patients with diabetes with signs of microangiopathy, the lung's diffusion capacity was significantly reduced [25]. In this cohort, compared with patients without diabetes, patients with diabetes with COVID-19 had significantly higher age and D-dimer. These factors might be involved in changing immune function and pulmonary function in patients with diabetes with COVID-19, which further promoted the patient's death. The mechanism needed further study.

However, with the small sample size of this retrospective study, selection bias might occur. This study was based on a single center, and a large-scale study was needed.

In conclusion, COVID-19 patients with diabetes had a significantly higher mortality (14.5%) than those without diabetes. Diabetes, higher levels of D-dimer, and lymphocyte count < 0.6 × 10^9^/L at admission were the risk factors for in-hospital death. Thus, COVID-19 patients with diabetes required extra attention.

## Figures and Tables

**Table 1:** Baseline characteristics of patients with COVID-19 pneumonia admission to the hospital.

		No. (%)		
	Total (*n* = 199)	Diabetic (*n* = 76)	Nondiabetic (*n* = 123)	*P* value
Age, median (IQR) (years)	63 (50-75)	67 (61-78)	59 (47-68)	≤0.001
Sex				
Female	110 (55.3)	37 (48.7)	73 (59.3)	0.142
Male	89 (44.7)	39 (51.3)	50 (40.7)	
Symptoms				
Fever	148 (74.4)	54 (71.1)	94 (76.4)	0.399
Cough	133 (66.8)	54 (71.1)	79 (64.2)	0.320
Dyspnea	53 (26.6)	22 (28.9)	31 (25.2)	0.562
Fatigue	50 (25.1)	16 (21.1)	34 (27.6)	0.289
Anorexia	32 (16.1)	9 (11.8)	23 (18.7)	0.201
Nausea	23 (11.6)	5 (6.6)	18 (14.6)	0.084
Headache	20 (10.1)	5 (6.6)	15 (12.2)	0.200
Diarrhea	6 (3.0)	1 (1.3)	5 (4.1)	0.410
Vomiting	5 (2.5)	1 (1.3)	3 (3.3)	0.651
Dizziness	2 (1.0)	1 (0.8)	1 (1.3)	>0.999
Onset of initial symptom to hospital admission (days) (IQR)	10 (6-15)	10 (5-14)	12 (7-20)	0.036
Duration of hospital stay (days) (IQR)	13(8-20)	13 (9-19)	12 (7-22)	0.968
Clinical outcome				
Died	18 (9.0)	11(14.5)	7(5.7)	0.036
Alive	181 (91.0)	65 (86.5)	116 (94.3)	

COVID-19: coronavirus disease 2019; IQR: interquartile range; No.: number.

**Table 2:** Laboratory findings of patients with COVID-19 at admission.

			Median (IQR)		*P* value
	Normal range	Total (*n* = 199)	Diabetic (*n* = 76)	Nondiabetic (*n* = 123)	
White blood cell count (×10^9^/L)	3.7-10	5.6 (4.1-7.0)	6.0 (4.4-7.9)	5.3 (4.1-6.5)	0.011
Lymphocyte count (×10^9^/L)	0.80-4.00	1.17 (0.80-1.61)	1.11 (0.73-1.42)	1.19 (0.84-1.73)	0.036
Prothrombin time (s)	9-13	12.2 (11.5-13.2)	12.3 (11.7-13.4)	12.2 (11.4-13.1)	0.430
Activated partial thromboplastin time (s)	20-40	26.4 (22.9-31.0)	25.8 (21.9-30.1)	26.7 (23-31.6)	0.105
D-dimer (mg/L)	0-0.55	0.62 (0.33-1.68)	0.96 (0.54-2.89)	0.50 (0.26-1.22)	≤0.001
Total bilirubin (mmol/L)	2-25	8.4 (5.9-13.2)	9.8 (7.0-14.6)	7.5 (5.8-11.2)	0.030
Albumin (g/L)	35-55	36.5 (33.5-40.2)	35.2 (31.9-38.3)	37.7 (34.7-40.7)	0.002
Blood urea nitrogen (mmol/L)	2.6-7.5	4.3 (3.3-6.5)	4.9 (3.5-7.8)	4.3 (3.3-5.5)	0.022
Creatinine (*μ*mol/L)	41-73	69.3 (55.1-83.5)	70.7 (55.3-84.0)	68.5 (55.1-83.5)	0.643
Serum potassium	3.5-5.5	3.77 (3.40-4.10)	3.75 (3.40-4.13)	3.77 (3.44-4.08)	0.587
Fasting plasma glucose	3.6-6.11	5.93 (5.03-7.80)	7.92 (6.54-10.19)	5.37(4.94-5.93)	≤0.001
Oxygen saturation	>93%	97% (95-98%)	96% (93-97%)	97% (95-98%)	≤0.001

COVID-19: coronavirus disease 2019; IQR: interquartile range; No.: number.

**Table 3:** Multivariable analysis of independent risk factors associated with in-hospital death.

Variables	*P* value	OR (95% CI)
Age, years	0.380	1.026 (0.969-1.087)
Cardiovascular disease (yes vs. no)	0.284	0.250 (0.020-3.155)
Chronic kidney disease (yes vs. no)	0.083	8.934 (0.752-106.073)
Hypertension (yes vs. no)	0.583	0.660 (0.150-2.908)
Diabetes (yes vs. no)	0.007	10.816 (1.895-61.741)
D-dimer at admission (mg/L)	0.016	1.094 (1.017-1.178)
Lymphocyte count at admission (<0.6 × 10^9^/L)	0.006	7.609 (1.807-32.049)
Fasting plasma glucose at admission	0.077	1.186 (0.982-1.432)
Treatment with low molecular weight heparin (yes vs. no)	0.330	0.418 (0.072-2.416)
Antidiabetic drugs (yes vs. no)	0.003	0.036 (0.004-0.317)

Antidiabetic drugs include oral drugs or/and insulin.

## Data Availability

The Excel data of this study can be obtained from the corresponding author upon request.
