# Erratum to “Clinical Characteristics of Diabetic Patients with COVID-19”

**DOI:** 10.1155/2021/9756140

**Published:** 2021-05-28

**Authors:** Guozhen Li, Qin Deng, Jiali Feng, Fang Li, Nian Xiong, Qiong He

**Affiliations:** ^1^Department of Gastroenterology, Wuhan Red Cross Hospital, Wuhan 430015, China; ^2^Department of Neurology, Union Hospital, Tongji Medical College, Huazhong University of Science and Technology, Wuhan 430022, China

Due to a production error, a version of the manuscript titled “Clinical Characteristics of Diabetic Patients with COVID-19” was published missing final editorial changes. The incorrect version of the manuscript is published alongside this Erratum as a supplementary file, and the original manuscript has been updated.

Hindawi apologises for causing this error in the article.

## Supplementary Materials

Supplementary Materials Incorrect version of the manuscript “Clinical Characteristics of Diabetic Patients with COVID-19”.
