# Supplementary material for: Erratum to “Clinical Characteristics of Diabetic Patients with COVID-19”
Source: J Diabetes Res. 2021 May 28;2021:9756140. doi: 10.1155/2021/9756140 (PMC8275428; doi:10.1155/2021/9756140)
Supplement: Supplementary Materials — Incorrect version of the manuscript “Clinical Characteristics of Diabetic Patients with COVID-19”. [file 9756140.f1.pdf]

Hindawi
Journal of Diabetes Research
Volume 2020, Article ID 1652403, 5 pages
https://doi.org/10.1155/2020/1652403

*Research Article*

# Clinical Characteristics of Diabetic Patients with COVID-19

**Guozhen Li,[1] Qin Deng,[1] Jiali Feng,[1] Fang Li,[1] Nian Xiong ,[1,2] and Qiong He [1]**

[1]*Department of Gastroenterology, Wuhan Red Cross Hospital, Wuhan 430015, China*
[2]*Department of Neurology, Union Hospital, Tongji Medical College, Huazhong University of Science and Technology, Wuhan 430022, China*

Correspondence should be addressed to Nian Xiong; nianxiong@hust.edu.cn and Qiong He; 13971470191@163.com

Received 7 April 2020; Revised 7 June 2020; Accepted 29 June 2020; Published 10 August 2020

Academic Editor: Janet H. Southerland

Copyright © 2020 Guozhen Li et al. This is an open access article distributed under the Creative Commons Attribution License, which permits unrestricted use, distribution, and reproduction in any medium, provided the original work is properly cited.

*Background*. Since December 2019, novel coronavirus- (SARS-CoV-2) infected pneumonia (COVID-19) has rapidly spread throughout China. This study is aimed at describing the characteristics of COVID-19 patients in Wuhan. *Methods*. 199 COVID-19 patients were admitted to Wuhan Red Cross Hospital in China from January 24th to March 15th. The cases were divided into diabetic and nondiabetic groups according to the history of taking antidiabetic drugs or by plasma fasting blood glucose level at admission, and the difference between groups were compared. *Results*. Among 199 COVID-19 patients, 76 were diabetic and 123 were nondiabetic. Compared with nondiabetics, patients with diabetes had an older age, high levels of fasting plasma glucose (FPG), D-dimer, white blood cell, blood urea nitrogen (BUN) and total bilirubin (TBIL), lower levels of lymphocyte, albumin and oxygen saturation ($SaO_2$), and higher mortality ($P < 0.05$). The two groups showed no difference in clinical symptoms. Diabetes, higher level of D-dimer at admission, and lymphocyte count less than $0.6 \times 10^9$/L at admission were associated with increasing odds of death. Antidiabetic drugs were associated with decreasing odds of death. Treatment with low molecular weight heparin was not related to odds of death. *Conclusion*. The mortality rate of COVID-19 patients with diabetes was significantly higher than those without diabetes. Diabetes, higher level of D-dimer, and lymphocyte count less than $0.6 \times 10^9$/L at admission were the risk factors associated with in-hospital death.

## 1. Introduction

Since December 2019, novel coronavirus- (SARS-CoV-2-) infected pneumonia (COVID-19) has rapidly spread throughout China and around the world [1–4]. The International Committee on Taxonomy of Viruses (ICTV) has named this virus SARS-CoV-2, with the disease termed COVID-19 [5]. The high infectivity of COVID-19 resulted in a rapid increase of new cases. Previous studies have described the epidemiological findings, clinical presentation, and clinical outcomes of patients with confirmed COVID-19 [6, 7]. However, specific information of patients with diabetes with COVID-19 remains unknown.

Diabetes mellitus (DM) is often identified as an independent risk factor for developing respiratory tract infections [8]. Studies have reported the relationship between blood glucose levels and the clinical course of severe acute respiratory syndrome (SARS) [9]. Up to now, information regarding the clinical characteristics of patients with diabetes with 2019 novel SARS-COV-2 pneumonia was scarce. In this study, the aim was to determine clinical symptoms, laboratory findings, and mortality of patients with diabetes and patients without diabetes in COVID-19, and to report on any difference.

## 2. Methods

*2.1. Patients*. The retrospective study was approved by the ethics committee of Wuhan Red Cross Hospital (No. 2020022). All patients with COVID-19 admitted to Wuhan Red Cross Hospital from January 24th to March 15th were enrolled. During this period, Wuhan Red Cross Hospital became a special designated hospital for the treatment of patients with COVID-19.

Table 1: Baseline characteristics of patients with COVID-19 pneumonia admission to hospital.

| | No. (%) Total ($n = 199$) | Diabetic ($n = 76$) | Nondiabetic ($n = 123$) | $P$ Value |
|---|---|---|---|---|
| Age, median (IQR), years | 63 (50-75) | 67 (61-78) | 59 (47-68) | ≤0.001 |
| Sex | | | | |
| Female | 110 (55.3) | 37 (48.7) | 73 (59.3) | 0.142 |
| Male | 89 (44.7) | 39 (51.3) | 50 (40.7) | |
| Symptoms | | | | |
| Fever | 148 (74.4) | 54 (71.1) | 94 (76.4) | 0.399 |
| Cough | 133 (66.8) | 54 (71.1) | 79 (64.2) | 0.320 |
| Dyspnea | 53 (26.6) | 22 (28.9) | 31 (25.2) | 0.562 |
| Fatigue | 50 (25.1) | 16 (21.1) | 34 (27.6) | 0.289 |
| Anorexia | 32 (16.1) | 9 (11.8) | 23 (18.7) | 0.201 |
| Nausea | 23 (11.6) | 5 (6.6) | 18 (14.6) | 0.084 |
| Headache | 20 (10.1) | 5 (6.6) | 15 (12.2) | 0.200 |
| Diarrhea | 6 (3.0) | 1 (1.3) | 5 (4.1) | 0.410 |
| Vomiting | 5 (2.5) | 1 (1.3) | 3 (3.3) | 0.651 |
| Dizziness | 2 (1.0) | 1 (0.8) | 1 (1.3) | >0.999 |
| Onset of initial symptom to hospital admission (days), (IQR) | 10 (6-15) | 10 (5-14) | 12 (7-20) | 0.036 |
| Duration of hospital stay (days), (IQR) | 13 (8-20) | 13 (9-19) | 12 (7-22) | 0.968 |
| Clinical outcome | | | | |
| Died | 18 (9.0) | 11 (14.5) | 7 (5.7) | 0.036 |
| Alive | 181 (91.0) | 65 (86.5) | 116 (94.3) | |

COVID-19: coronavirus disease 2019; IQR: interquartile range; no: number.

*2.2. Definitions.* COVID-19 was confirmed by detecting SARS-CoV-2 RNA in throat swab samples using a virus nucleic acid detection kit according to the manufacturer's protocol (Shanghai BioGerm Medical Biotechnology Co., Ltd). All of patients were admitted to the general fever ward excluding the intensive care unit. The cases were divided into diabetic and nondiabetic groups according to the history of taking antidiabetic drugs or by plasma fasting blood glucose level at admission.

*2.3. Data Collection.* The case report form of COVID-19 was designed to document primary clinical data regarding previous medical history, clinical symptoms, laboratory findings, and clinical outcomes from electronic medical records. The following information was extracted for each patient: gender, age, medical history, clinical outcomes, and signs, symptoms, oxygen saturation, and laboratory findings at admission.

*2.4. Data Analysis.* Categorical data were described as percentages, and continuous data as median with interquartile range (IQR). $\chi^2$ test for categorical data and Mann-Whitney $U$ test for continuous data were used to compare variables between groups. All statistical analyses were performed using SPSS Statistics version 16.0 software. $P < 0.05$ was considered statistically significant.

## 3. Results

*3.1. Baseline Characteristics.* 199 patients with COVID-19 pneumonia were included in our study (Table 1). Among them, 76 were diabetic and 123 were nondiabetic. The median age of patients with COVID-19 pneumonia with diabetes was 67 years (IQR: 61~78), which was significantly higher than that of patients without diabetes (IQR: 47~68, $P \leq 0.001$). The onset of initial symptom to hospital admission in patients with diabetes group was 10 days (IQR: 5~14), while patients without diabetes group was 12 days (IQR: 7~20, $P = 0.036$). Duration of hospital stays was similar for both groups ($P = 0.968$). Of 199 patients, fever (74.4%), cough (66.8%), dyspnea (26.8%), and fatigue (25.1%) were the most common symptoms, while diarrhea (3%), vomiting (2.5%), and dizziness (1%) were scarce. However, no statistically significant difference in all of clinical symptoms between diabetic and nondiabetic patients with COVID-19 pneumonia.

*3.2. Laboratory Findings.* The majority of patients with diabetes had abnormalities of D-dimer and fasting plasma glucose (FPG) at admission as described in Table 2. Compared with patients without diabetes, patients with diabetes had a higher level of fasting plasma glucose ($P \leq 0.001$), D-dimer ($P \leq 0.001$), white blood cell ($P = 0.011$), total bilirubin ($P = 0.030$), and blood urea nitrogen ($P = 0.022$), and lower

Table 2: Laboratory findings of patients with COVID-19 at admission.

| | Normal range | Median (IQR) Total ($n = 199$) | Diabetic ($n = 76$) | Nondiabetic ($n = 123$) | $P$ value |
|---|---|---|---|---|---|
| White blood cell count, $\times 10^9$/L | 3.7-10 | 5.6 (4.1-7.0) | 6.0 (4.4-7.9) | 5.3 (4.1-6.5) | 0.011 |
| Lymphocyte count, $\times 10^9$/L | 0.80-4.00 | 1.17 (0.80-1.61) | 1.11 (0.73-1.42) | 1.19 (0.84-1.73) | 0.036 |
| Prothrombin time, s | 9-13 | 12.2 (11.5-13.2) | 12.3 (11.7-13.4) | 12.2 (11.4-13.1) | 0.430 |
| Activated partial thromboplastin time, s | 20-40 | 26.4 (22.9-31.0) | 25.8 (21.9-30.1) | 26.7 (23-31.6) | 0.105 |
| D-dimer, mg/L | 0-0.55 | 0.62 (0.33-1.68) | 0.96 (0.54-2.89) | 0.50 (0.26-1.22) | ≤0.001 |
| Total bilirubin, mmol/L | 2-25 | 8.4 (5.9-13.2) | 9.8 (7.0-14.6) | 7.5 (5.8-11.2) | 0.030 |
| Albumin, g/L | 35-55 | 36.5 (33.5-40.2) | 35.2 (31.9-38.3) | 37.7 (34.7-40.7) | 0.002 |
| Blood urea nitrogen, mmol/L | 2.6-7.5 | 4.3 (3.3-6.5) | 4.9 (3.5-7.8) | 4.3 (3.3-5.5) | 0.022 |
| Creatinine, $\mu$mol/L | 41-73 | 69.3 (55.1-83.5) | 70.7 (55.3-84.0) | 68.5 (55.1-83.5) | 0.643 |
| Serum potassium | 3.5-5.5 | 3.77 (3.40-4.10) | 3.75 (3.40-4.13) | 3.77 (3.44-4.08) | 0.587 |
| Fasting plasma glucose | 3.6-6.11 | 5.93 (5.03-7.80) | 7.92 (6.54-10.19) | 5.37(4.94-5.93) | ≤0.001 |
| Oxygen saturation | >93% | 97% (95-98%) | 96% (93-97%) | 97% (95-98%) | ≤0.001 |

COVID-19, coronavirus disease 2019; IQR, interquartile range; no, number.

Table 3: Multivariate analysis of independent risk factors associated with in-hospital death.

| Variables | $P$ value | OR (95% CI) |
|---|---|---|
| Age, years | 0.380 | 1.026 (0.969-1.087) |
| Cardiovascular disease (yes versus no) | 0.284 | 0.250 (0.020-3.155) |
| Chronic kidney disease (yes versus no) | 0.083 | 8.934 (0.752-106.073) |
| Hypertension (yes versus no) | 0.583 | 0.660 (0.150-2.908) |
| Diabetes (yes versus no) | 0.007 | 10.816 (1.895-61.741) |
| D-dimer at admission, mg/L | 0.016 | 1.094 (1.017-1.178) |
| Lymphocyte count at admission $(<0.6 \times 10^9/\mathrm{L})$ | 0.006 | 7.609 (1.807-32.049) |
| Fasting plasma glucose at admission | 0.077 | 1.186 (0.982-1.432) |
| Treatment with low molecular weight heparin (yes versus no) | 0.330 | 0.418 (0.072-2.416) |
| Antidiabetic drugs (yes versus no) | 0.003 | 0.036 (0.004-0.317) |

Antidiabetic drugs include oral drugs or/and insulin.

level of oxygen saturation ($P \leq 0.001$), lymphocyte ($P = 0.036$), and albumin ($P = 0.002$).

*3.3. Regression Analysis.* We included 199 patients with complete data for all variables (181 survivors and 18 nonsurvivors) in the multivariable logistic regression model. We found that diabetes, higher level of D-dimer at admission, and lymphocyte count less than $0.6 \times 10^9$/L at admission were associated with increasing odds of death. Antidiabetic drugs were associated with decreasing odds of death. Treatment with low molecular weight heparin was not related to odds of death (Table 3).

*3.4. Clinical Outcome.* There were 18 reported deaths in the COVID-19 patients (Table 1). Significant difference in mortality was found between patients with diabetes and without diabetes ($P = 0.036$). 11 of 76 patients with COVID-19 with diabetes died (14.5%), while 7 of 123 patients with COVID-19 without diabetes died (5.7%). Diabetes seems to increase the risk of death in patients with COVID-19 pneumonia (Log rank $P = 0.031$) (Figure 1).

## 4. Discussion

Coronavirus has received more attention compared to other causes of pneumonia, especially after the emergence of SARS and MERS. In certain risk factors, clinical manifestations, and clinical outcomes, COVID-19 was similar to SARS and MERS. It had been reported that a known history of diabetes was independent predictors for morbidity and death in patients with SARS [9]. In our study, 11 (14.5%) patients with COVID-19 pneumonia with diabetes died, while 7 (5.7%) patients with COVID-19 pneumonia without diabetes died ($P = 0.036$). Diabetes was associated with increasing odds of death. Antidiabetic drugs were associated with decreasing odds of death. Until now, large-scale analyses of clinical characteristics and outcome of patients with COVID-19 pneumonia with diabetes had been scarce. In this study, 199 COVID-19 patients were divided into diabetic and nondiabetic groups. We compared the clinical features, laboratory findings, and clinical outcome between the two groups.

The study found similar proportions of male and female patients in COVID-19 with and without diabetes.

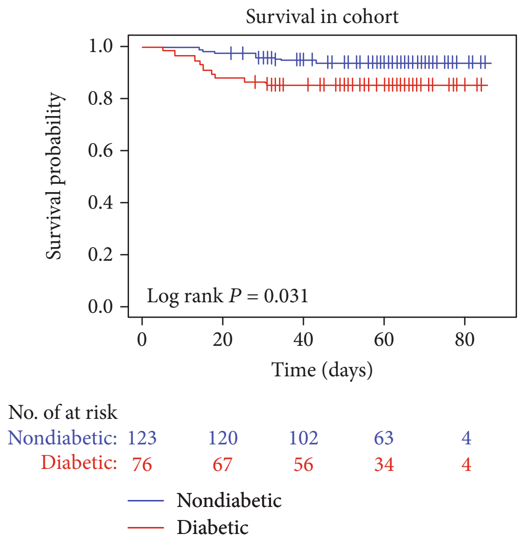


Figure 1: Survival curve in patients with COVID-19 pneumonia with and without diabetes.

The median age of patients with diabetes with COVID-19 was significantly older than that of patients without diabetes. According to previous reports, older age was an important independent predictor of mortality in MERS and SARS [10, 11]. Recent studies had confirmed that death in patients with COVID-19 was associated with increased age [12]. Nearly 85% of COVID-19-related death in Italy have been individuals in the 70+ year-old age group. It is common sense that in a patient with a fatality, in the presence of severe renal or pulmonary or cardiac disease, the mere presence of COVID-19 positivity does not confirm the role of coronavirus in causing death [13]. In our study, patients with diabetes with COVID-19 were older than patients without diabetes. Therefore, they were at increased risk for death also for their age.

In our cohort, compared with nondiabetic patients, patients with diabetes had a lower level of lymphocyte. Lymphocyte count less than $0.6 \times 10^9$/L at admission was associated with increasing odds of death. In previous studies, lymphocytopenia is also common in the critically ill patients with MERS infection, which is the result of apoptosis of lymphocytes [14, 15]. Yang et al. reported that lymphocytopenia occurred in more than 80% of critically ill patients with COVID-19 [16]. Lymphocytopenia is a prominent feature of critically ill patients with COVID-19 because targeted invasion by SARS-CoV viral particles damages the cytoplasmic component of the lymphocyte and causes its destruction [17]. Hence, we speculate that necrosis or apoptosis of lymphocytes also induces lymphocytopenia in critically ill patients with COVID-19. In our study, patients with diabetes with COVID-19 at admission had only mild lymphocytopenia. The severity of lymphocytopenia may reflect the aggravation of the disease.

Higher level of D-dimer at admission was associated with increased odds of death in our study. D-dimer levels were quite different between the diabetic and nondiabetic groups ($P \leq 0.001$). For patients with diabetes with COVID-19, D-dimer levels increased dramatically. D-dimer is an activation marker of fibrinolysis. Some studies have shown that D-dimer is a significant prognostic factor in patients with pneumonia and sepsis [18, 19]. D-dimer is a marker of mortality in patients admitted to the emergency department with suspected infection and sepsis [19]. In recent studies, the level of D-dimer was higher in the death group than in the survival group, and elevated levels of D-dimer at admission were risk factors for death of adult patients with COVID-19 [7, 12, 20]. We found that patients with COVID-19 with higher level of D-dimer at admission, especially those with diabetes, are significantly associated with the risk of death. Treatment with low molecular weight heparin was not related to odds of death. Magro et al. reported that severe COVID-19 infection was associated with microvascular injury and thrombosis [21]. There is a need for further clinical trials using anticoagulants to determine whether the application of anticoagulants is effective.

Currently, few public studies have shown the specific cause of high mortality in patients with COVID-19 with diabetes. Diabetes mellitus (DM) has been identified as an independent risk factor for developing respiratory infections. Many changes occurred in the immune system of DM patients. There were significant changes in humoral and cell-mediated immune function, especially related to abnormal pulmonary function.

In patients with diabetes with signs of microangiopathy, the lung's diffusion capacity was significantly reduced [22]. In this cohort, compared with patients without diabetes, patients with diabetes with COVID-19 had significantly higher age and D-dimer. These factors might be involved in changing immune function and pulmonary function in patients with diabetes with COVID-19, which further promoted the patient's death. The mechanism needed further study.

However, with the small sample size of this retrospective study, selection bias might occur. This study was based on a single center, and a large-scale study was needed.

In conclusion, the mortality rate for diabetic patients with COVID-19 was 14.5%, which was significantly higher than that of patients without diabetes. Diabetes, higher level of D-dimer, and lymphocyte count less than $0.6 \times 10^9$/L at admission were the risk factors associated with in-hospital death. So, patients with COVID-19 with diabetes require extra attention.

## Data Availability

The Excel data used to support the findings of this study are available from the corresponding author upon request.

## Conflicts of Interest

The authors declare that they have no conflicts of interest.

## Authors' Contributions

GZL, QD, JLF, and FL collected the data. GZL, NX, and HQ prepared and revised the manuscript. GZL, NX, and QH were responsible for summarizing all data related to this study. Guozhen Li and Qin Deng contributed equally to this work.

## Acknowledgments

This work was supported by grants 2016YFC1306600 (to NX) and 2018YFC1314700 (to NX) from the National Key R&D Program of China and grant 81873782 (to NX) from the National Natural Science Foundation of China.

## References

[1] N. Zhu, D. Zhang, W. Wang et al., "A novel coronavirus from patients with pneumonia in China, 2019," *The New England Journal of Medicine*, vol. 382, no. 8, pp. 727–733, 2020.

[2] M. L. Holshue, C. DeBolt, S. Lindquist et al., "First case of 2019 novel coronavirus in the United States," *The New England Journal of Medicine*, vol. 382, no. 10, pp. 929–936, 2020.

[3] C. Huang, Y. Wang, X. Li et al., "Clinical features of patients infected with 2019 novel coronavirus in Wuhan, China," *Lancet*, vol. 395, no. 10223, pp. 497–506, 2020.

[4] Q. Li, X. Guan, P. Wu et al., "Early transmission dynamics in Wuhan, China, of novel coronavirus-infected pneumonia," *The New England Journal of Medicine*, vol. 382, no. 13, pp. 1199–1207, 2020.

[5] H. A. Rothan and S. N. Byrareddy, "The epidemiology and pathogenesis of coronavirus disease (COVID-19) outbreak," *Journal of Autoimmunity*, vol. 109, p. 102433, 2020.

[6] N. Chen, M. Zhou, X. Dong et al., "Epidemiological and clinical characteristics of 99 cases of 2019 novel coronavirus pneumonia in Wuhan, China: a descriptive study," *Lancet*, vol. 395, no. 10223, pp. 507–513, 2020.

[7] D. Wang, B. Hu, C. Hu et al., "Clinical characteristics of 138 hospitalized patients with 2019 novel coronavirus-infected pneumonia in Wuhan, China," *JAMA*, vol. 323, no. 11, p. 1061, 2020.

[8] R. B. Klekotka, E. Mizgała, and W. Król, "The etiology of lower respiratory tract infections in people with diabetes," *Pneumonologia i Alergologia Polska*, vol. 83, no. 5, pp. 401–408, 2015.

[9] J. K. Yang, Y. Feng, M. Y. Yuan et al., "Plasma glucose levels and diabetes are independent predictors for mortality and morbidity in patients with SARS," *Diabetic Medicine*, vol. 23, no. 6, pp. 623–628, 2006.

[10] K. W. Choi, T. N. Chau, O. Tsang et al., "Outcomes and prognostic factors in 267 patients with severe acute respiratory syndrome in Hong Kong," *Annals of Internal Medicine*, vol. 139, no. 9, pp. 715–723, 2003.

[11] K. H. Hong, J. P. Choi, S. H. Hong et al., "Predictors of mortality in Middle East respiratory syndrome (MERS)," *Thorax*, vol. 73, no. 3, pp. 286–289, 2018.

[12] F. Zhou, T. Yu, R. du et al., "Clinical course and risk factors for mortality of adult inpatients with COVID-19 in Wuhan, China: a retrospective cohort study," *Lancet*, vol. 395, no. 10229, pp. 1054–1062, 2020.

[13] S. S. Chakrabarti, U. Kaur, A. Banerjee et al., "COVID-19 in India: are biological and environmental factors helping to stem the incidence and severity?," *Aging and Disease*, vol. 11, no. 3, pp. 480–488, 2020.

[14] H. Chu, J. Zhou, B. H. Y. Wong et al., "Middle East respiratory syndrome coronavirus efficiently infects human primary T lymphocytes and activates the extrinsic and intrinsic apoptosis pathways," *The Journal of Infectious Diseases*, vol. 213, no. 6, pp. 904–914, 2016.

[15] W. J. Liu, M. Zhao, K. Liu et al., "T-cell immunity of SARS-CoV: implications for vaccine development against MERS-CoV," *Antiviral Research*, vol. 137, pp. 82–92, 2017.

[16] X. Yang, Y. Yu, J. Xu et al., "Clinical course and outcomes of critically ill patients with SARS-CoV-2 pneumonia in Wuhan, China: a single-centered, retrospective, observational study," *The Lancet Respiratory Medicine*, vol. 8, no. 5, pp. 475–481, 2020.

[17] J. Gu, E. Gong, B. Zhang et al., "Multiple organ infection and the pathogenesis of SARS," *The Journal of Experimental Medicine*, vol. 202, no. 3, pp. 415–424, 2005.

[18] E. B. Milbrandt, GenIMS Investigators, M. C. Reade et al., "Prevalence and significance of coagulation abnormalities in community-acquired pneumonia," *Molecular Medicine*, vol. 15, no. 11-12, pp. 438–445, 2009.

[19] J. R. Rodelo, G. de la Rosa, M. L. Valencia et al., "D-dimer is a significant prognostic factor in patients with suspected infection and sepsis," *The American Journal of Emergency Medicine*, vol. 30, no. 9, pp. 1991–1999, 2012.

[20] C. Wu, X. Chen, Y. Cai et al., "Risk factors associated with acute respiratory distress syndrome and death in patients with coronavirus disease 2019 Pneumonia in Wuhan, China," *JAMA Internal Medicine*, vol. 180, no. 7, pp. 1–11, 2020.

[21] C. Magro, J. J. Mulvey, D. Berlin et al., "Complement associated microvascular injury and thrombosis in the pathogenesis of severe COVID-19 infection: a report of five cases," *Translational Research*, vol. 220, no. 20, pp. 1–13, 2020.

[22] N. Joshi, G. M. Caputo, M. R. Weitekamp, and A. W. Karchmer, "Infections in patients with diabetes mellitus," *The New England Journal of Medicine*, vol. 341, no. 25, pp. 1906–1912, 1999.
